# HCV coinfection aggravated the decrease of platelet counts, but not mean platelet volume in chronic HIV-infected patients

**DOI:** 10.1038/s41598-018-35705-9

**Published:** 2018-11-30

**Authors:** Linting Lv, Yuantao Li, Xueying Fan, Zhe Xie, Hua Liang, Tao Shen

**Affiliations:** 1 Department of Microbiology and Center of Infectious Disease, School of Basic Medical Sciences, Peking University Health Science Center, Beijing, 100191 China; 2 State Key laboratory of Infectious Disease Prevention and Control (SKLID), National Center for AIDS/STD Control and Prevention, China CDC, Collaborative Innovation Center for Diagnosis and Treatment of Infectious Diseases, Beijing, 102206 China

## Abstract

Either HIV or HCV monoinfection could result in an abnormal status of platelets. As two key indicators reflecting activation and function of platelets, the changes of platelet counts and mean platelet volume (MPV) in HIV/HCV-coinfected patients have not been clearly identified. In the present study, a total of 318 former plasma donors were investigated in 2006, and 66% (201 individuals) of primary recruiters were followed up in 2014. By horizontal comparison in 2006, the decrease of platelet counts in HIV/HCV coinfection was greater than that in HIV or HCV monoinfection. MPV scores were lower in HIV monoinfection compared with healthy controls, while no difference was found in HIV/HCV coinfection. Platelet counts were shown to be negatively correlated with MPV scores in total recruited population (*r* = 0.432, *P* < 0.001). Interestingly, by comparison of data from two time points of 2006 and 2014, significant decrease of platelets (*P* = 0.004) and increase of MPV (*P* = 0.004) were found only in HCV monoinfected patients, which may associate with slow progression of hepatic fibrosis induced by chronic HCV infection. Nonetheless, no significant changes of platelet counts and MPV were found from 2006 to 2014 in coinfected patients. In conclusion, HCV coinfection aggravated the decrease of platelet counts, but not MPV score in chronic HIV infection. MPV showed poor applicability in reflecting the status of platelets in HIV/HCV-coinfected patients.

## Introduction

Thrombocytopenia is a common syndrome of human immunodeficiency virus-1 (HIV-1)-infection^1^, which may ascribe to malignancies, medications, secondary infection, platelet antibody-mediated phagocytosis by macrophages, and possible a direct effect of HIV-1 on platelet production^2,3^. In addition, the role of platelets in inflammation, chronic immune activation and microvascular dysfunction during chronic HIV infection has also been frequently described^4–11^.

The alternations of platelet count and mean platelet volume (MPV), two key indicators reflecting activation and function of platelets, have been investigated in HIV infection^12–14^. Platelet activation might suppress HIV-1 infection of T cells and platelet counts were found to be inversely related to plasma HIV-1 RNA levels^11,14^. Approximate 10%~50% of HIV-infected individuals were reported to suffer from thrombocytopenia^15^, among which more than half experienced a platelet count less than 50 × 10^9^/L with more than one-third experiencing a nadir below 30 × 10^9^/L^16^. MPV is a typical laboratory marker for measurement of platelet size. Since platelets will shrink after becoming senescent, decreased MPV score is generally associated with weakened hematopoietic function of bone marrow whereas high MPV usually means high destruction of platelets such as immune thrombocytopenic purpura and sepsis^17–19^. MPV is widely used as an inflammatory marker to evaluate platelet activity and status of systemic inflammation. Large size of platelet has been correlated with higher activation, aggregation, thromboxane synthesis and beta-thromboglobulin release^20,21^. Higher MPV might indicate an increased risk of acute cardiovascular diseases (CVD). Some reports showed that elevated MPV was correlated with a worse outcome in patients with some CVD and cerebrovascular diseases, such as acute stroke, myocardial infarction, and acute coronary syndromes^13,22–24^. CVD is a common complication of HIV-1 infection, which may shorten the lifespan of affected patients^25–27^. Likewise, chronic hepatitis C virus (HCV) infection is also associated with extrahepatic complications, including CVD^28,29^. Whereas, several studies showed that MPV is decreased in HIV-infected patients^30,31^, suggesting impaired platelet production rather than increased destruction. In HCV infection, the status of platelet was closely correlated with degree of fibrosis, platelet counts was decreased while MPV was increased in chronic hepatitis C (CHC) patients, especially in those with advanced fibrosis^32,33^.

Coinfection with hepatitis C virus is common in HIV-infected patients due to shared routes of viral transmission^34–36^. It is estimated that approximately 25% to 33% of HIV/AIDS patients were infected with HCV^37^. Both of chronic HIV and HCV infection are associated with platelet disorders, although their features and underlying mechanisms are differed from two type of viral infection. However, few studies have focused on the manifestation of platelets in HIV/HCV-coinfected patients. We hypothesized that a synergistical effect of platelet disorders induced by HIV and HCV infection would present in those coinfected patients. The aim of this study was to explore the features of platelet counts and MPV in the case of HIV/HCV coinfection.

## Results

### Comparison of platelet counts and MPV scores among patients infected with HCV and/or HIV

**Figure 1:**
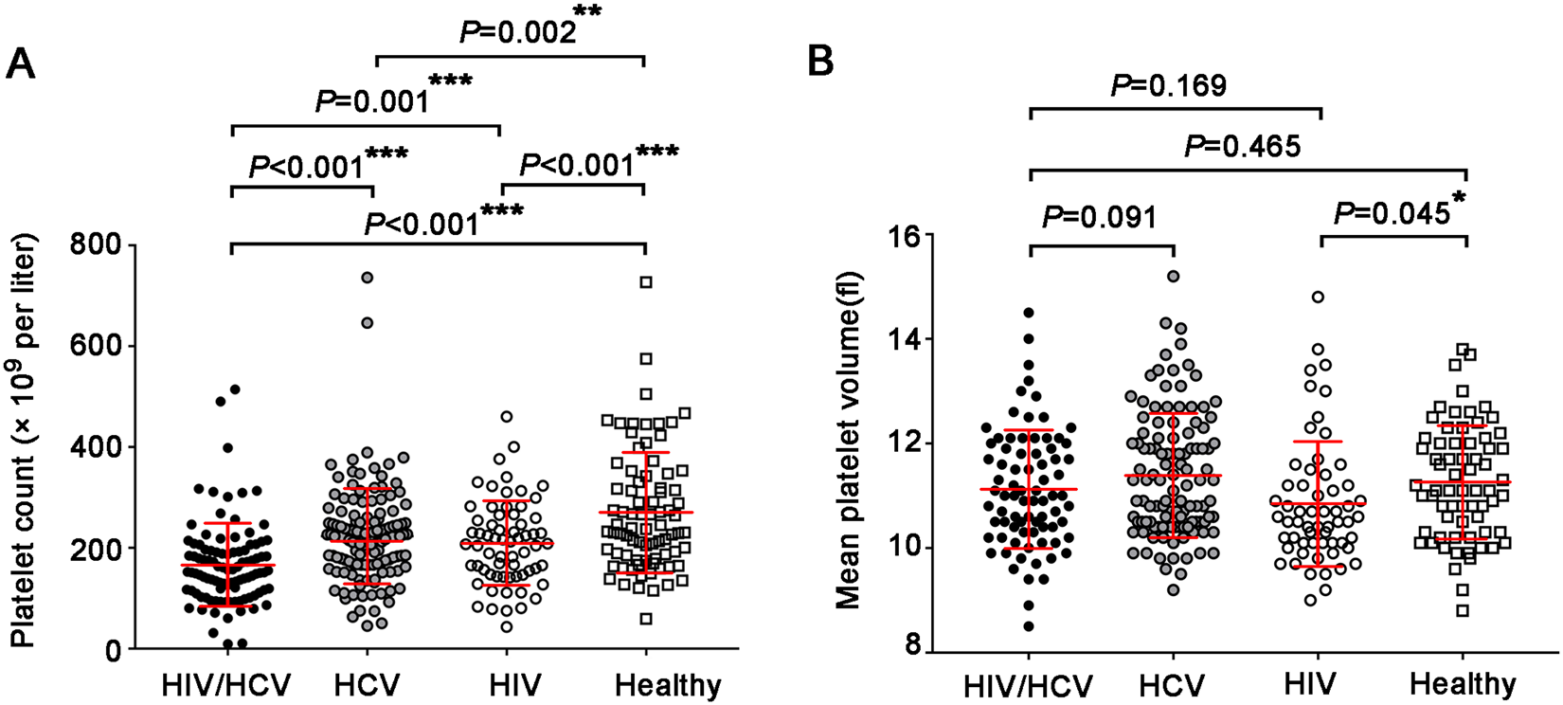
Comparison of platelet counts (**A**) and mean platelet volume (MPV) scores (**B**) among patients infected with HCV and/or HIV and healthy controls. All data were shown as mean ± standard deviation (SD) and asterisks indicated significant levels by the Mann-Whitney U test (2-tailed; *P < 0.05; **P < 0.01, ***P < 0.001).

In this study, 318 participants recruited in 2006 were divided into 4 groups (HIV/HCV coinfection, HCV momoinfection, HIV monoinfection and healthy controls) depending on the existence of anti-HCV, HCV RNA and anti-HIV. As expected, platelet counts in patients infected with HIV and/or HCV were lower than that in healthy controls (*P* < 0.001 for HIV/HCV or HIV vs. healthy; *P* = 0.002 for HCV vs. healthy). Specially, HIV/HCV-coinfected patients exhibited much lower platelet counts than HCV (*P* < 0.001) or HIV monoinfected subjects (*P* = 0.001) (Fig. 1A). However, no differences were found in MPV between HIV/HCV coinfection and HCV or HIV monoinfection, or healthy individuals, in spite of lower MPV was found in HIV group compared with healthy controls (*P* = 0.045) (Fig. 1B). Taken together, these data indicated that HCV coinfection may aggravate the decrease of platelet count in chronic HIV-infected patients, and also HIV infection would accelerate the decrease of platelet count induced by HCV infection. However, HCV coinfection did not aggravate, and even partially recover the decrease of MPV induced by HIV infection.

### Low CD4+ T-cells and high ALT levels were associated with decreased platelet counts

**Figure 2:**
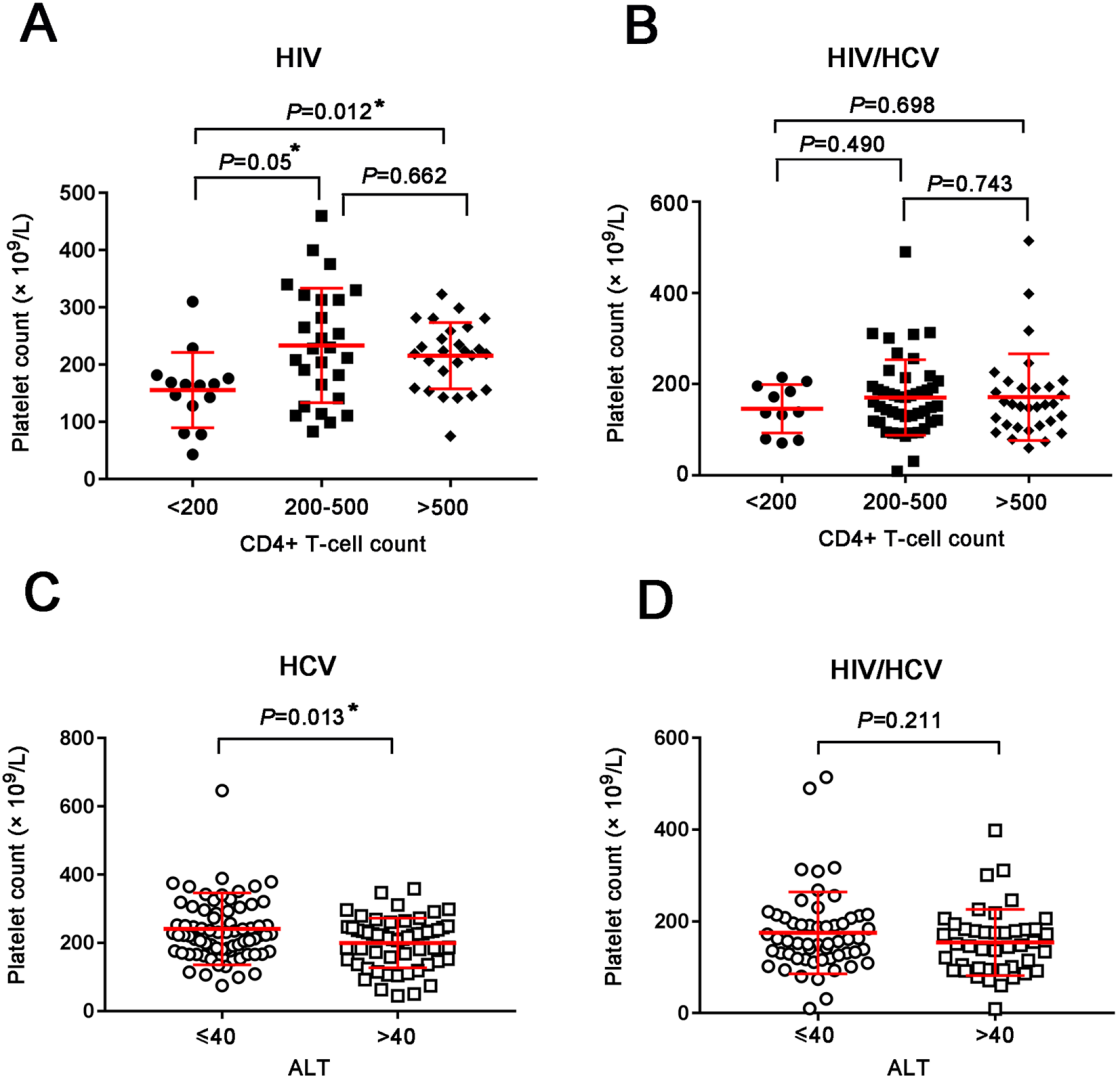
Comparison of platelet counts among different subgroups according to CD4+ T-cell counts and ALT level. HIV (**A**) and HIV/HCV (**B**) groups were divided into three subgroups according to CD4+ T-cell count (<200/μl, 200–500/μl and >500/μl). Similarly, HCV (**C**) and HIV/HCV (**D**) groups were divided into two subgroups according to serum ALT level (≤40 IU/L and >40 IU/L). All data were shown as mean ± standard deviation (SD) and asterisks indicated significant levels by the Mann-Whitney U test (2-tailed; *P < 0.05; **P < 0.01, ***P < 0.001).

In HIV-monoinfected patients, platelet counts in subgroup with low CD4^+^ T-cells (<200/μl) were significantly lower than patients with CD4^+^ T-cells within 200~500/μl (*P* = 0.05), and those with CD4^+^ T-cells higher than 500/μl (*P* = 0.012) (Fig. 2A). However, no similar differences were found in HIV/HCV-coinfected patients, which might ascribe to the complicated condition caused by HCV coinfection (Fig. 2B). The level of serum alanine transaminase (ALT) is commonly measured clinically as a biomarker for liver function. Considering that platelet counts may be related to hepatic inflammation in HCV infection, we further compared PLT counts between patients with normal ALT (≤40 IU/L) and high ALT (>40 IU/L) both in HCV-monoinfected and HIV/HCV-coinfected patients. The results indicated that lower PLT counts were only found in HCV-monoinfection (*P* = 0.013), but not in HIV/HCV coinfection (Fig. 2C,D).

Unfortunately, we failed to find any differences of MPV between subgroups divided by CD4+ T-cell counts or ALT level in monoinfected or coinfected groups in this study (Fig. S1).

### Platelet counts were negatively correlated with MPV in total recruited subjects and subpopulations of four groups

**Figure 3:**
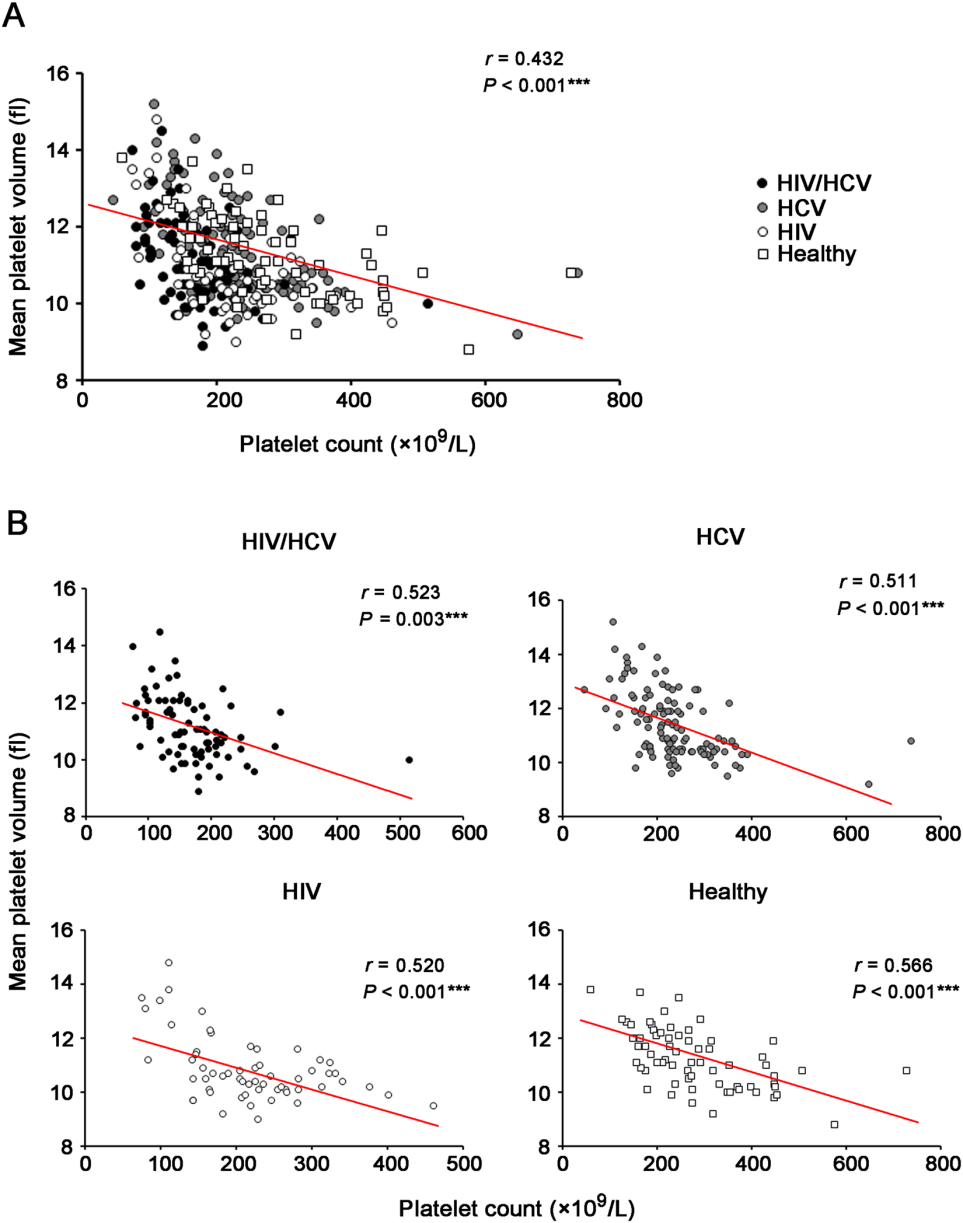
Correlation analysis between platelet counts and mean platelet volume (MPV) in total 318 recruited individuals (**A**) and four individual groups (**B**) based on the status of HCV and HIV infection. Asterisks indicated significant levels by the Spearman’s rank-correlation test (2-tailed; ***P < 0.001).

The correlation between platelet counts and MPV was analyzed (Fig. 3A). Negative correlations between these two indicators were found not only in all 318 initially recruited individuals (*r* = 0.432, *P* < 0.001, Fig. 3A), but also in four subpopulations (*r* = 0.523, *P* = 0.003 for HIV/HCV coinfection; *r* = 0.511, *P* < 0.001 for HCV monoinfection; *r* = 0.520, *P* < 0.001 for HIV monoinfection; *r* = 0.566, *P* < 0.001 for healthy controls) (Fig. 3B). These finding indicated that, although infection of HCV and/or HIV might change the count or size of platelets, the correlation between these two indexes always existed.

### A longitudinal dynamic analysis of platelet counts and MPV from 2006 to 2014

**Figure 4:**
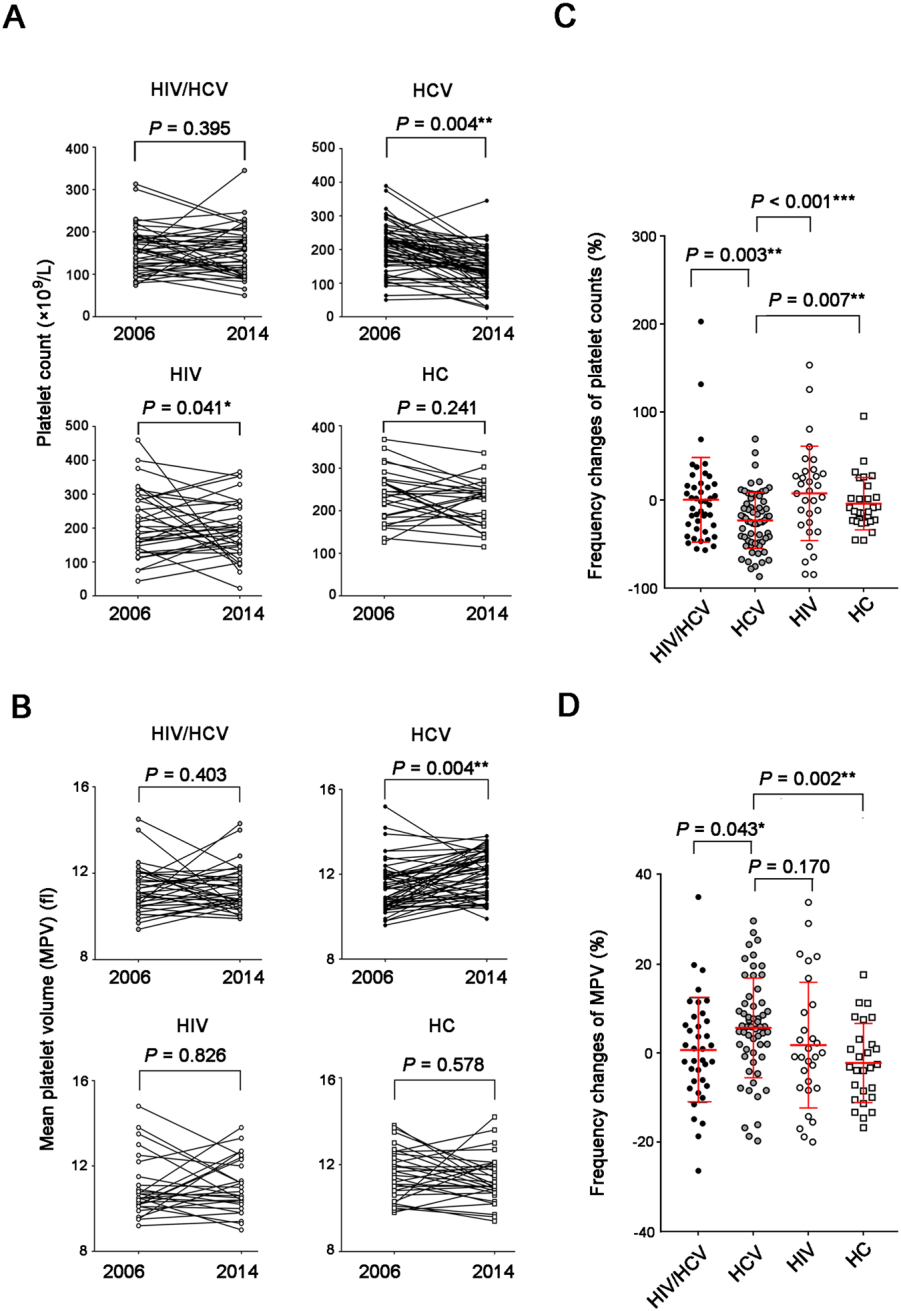
A longitudinal dynamic analysis of platelet counts and MPV from 2006 to 2014. Comparison of platelet counts (**A**) and MPV (**B**) scores in 2006 (primary visit) and 2014 (follow-up visit) in four different groups based on the status of HCV and HIV infection. In addition, the decreasing rates of PLT counts (**C**) and the increasing rates of MPV (**D**) among these four groups were compared respectively. Comparisons between groups were performed using Wilcoxon matched-pairs (**A**,**B**) and Mann-Whitney U-tests (**C**,**D**). All p-values were considered significant when lower than 0.05 (2-tailed; *P < 0.05; **P < 0.01, ***P < 0.001).

By comparison of platelet counts and MPV in samples collected at two different time points in 2006 and 2014, we found a significant decrease of platelet (*P* = 0.004) and increase of MPV (*P* = 0.004) in HCV monoinfected patients (Fig. 4A,B). Platelet counts had decreased a little from 2006 to 2014 in HIV monoinfected group (*P* = 0.041) (Fig. 4A). In addition, we analyzed the decreasing or increasing rate of platelet counts and MPV from 2006 to 2014 in each individual group. As shown in Fig. 4C,D, the decreasing rate of PLT counts (*P* = 0.003 for HIV/HCV vs. HCV; *P* < 0.001 for HCV vs. HIV; *P* = 0.007 for HCV vs. HC) (Fig. 4C) and the increasing rate of MPV (*P* = 0.043 for HIV/HCV vs. HCV; *P* = 0.002 for HCV vs. HC) (Fig. 4D) in HCV-monoinfected patients were significantly higher than the other three groups. However, no significant changes were found in coinfected patients from 2006 to 2014.

In addition, we failed to find any associations between the level of PLT or MPV in 2006 with different clinical prognosis of anti-HIV therapy in 2014 in all HIV-infected patients (Table S3). This result may indicated that, although the decrease of PLT was observed in HIV infection and aggravated by HCV coinfection, it is still indeterminable whether the levels of platelet indexes could be used as an useful predicator for evaluating the effect of anti-HIV therapy.

## Discussion

With the availability of antiretroviral therapy (ART), HIV-1 infection is now considered as a manageable chronic disease. Morbidity and mortality of HIV-related opportunistic diseases declined and the lifespan and living quality of the HIV/AIDS cases are dramatically improved^38^. On the other hand, there is an increased susceptibility to developing CVD in these patients. Coinfection of HCV would aggravate the persistent inflammatory response and immune activation and then synergistically increase the risk of CVD^9,12,17,39^. As the key indicators of CVD risk in complete blood count analysis, it should be helpful to analyze the features of platelet counts and MPV for identifying risk of CVD in HIV/HIV-coinfected patients.

Our data showed that lower platelet counts were found in patients with lower CD4^+^ T-cell (<200/μl), either in HIV/HCV coinfection or in HIV monoinfection. Previous study pointed out CD4+ T cells in blood of HIV-infected patients were skewed toward a monofunctional Th1 phenotype with cellular maturation^40^. Although the mechanism by which Th1 cells affect platelets is still unclear, it is widely believed that they are closely linked, for example the high Th1/Th2 ratio was closely related to the etiology and disease status of chronic idiopathic thrombocytopenic purpura(ITP)^41,42^. Th1 activation is likely to induce local inflammation with concomitant secretion of pro-inflammatory and macrophage-associated cytokines and it dominates among plaque-infiltrating lymphocytes^43^. There are many direct evidence strongly support the notion that a Th1-type response takes place in the atherosclerotic plaque^44,45^, which can partially explain the high risk of cardiovascular disease in HIV. In HIV/HCV coinfection, HCV RNA^+^ individuals had significantly slower recovery of CD4^+^ T-cell on antiretroviral therapy (ART) compared with HCV RNA^–^individuals (on average 7 times lower)^46^. On the other hand, many studies showed that both ALT and platelet counts correlated with fibrosis stage in patients without a history of excessive alcohol use^47,48^. The prevalence of thrombocytopenia increased remarkably along with severity of liver disease among participants with HCV infection. Meanwhile, an elevated serum ALT level was strongly associated with thrombocytopenia^49,50^. Consistently, our study also found that platelet counts in subgroup with higher ALT were lower than patients with normal ALT in HCV-monoinfected patients.

We hypothesized HIV infection would result in reduction of platelet counts and MPV score, which might occur in the relatively early stage of HIV infection. Our study showed that, compared to the data of 2006, the values of platelet counts and MPV in 2014 did not decrease greatly in HIV-monoinfected group, which suggested that these two indicators were already very low in 2006. Different with HIV infection, the changes of platelet counts and MPV score induced by chronic HCV infection appeared as a gradually slow progression. The 8-year follow-up investigation showed that these two platelet indicators had changed significantly (*P* < 0.001 for both) in 2014 compared to the primary visit in 2006. This feature of changes may associate with gradual development from long-term inflammation to hepatic fibrosis and even cirrhosis in chronic HCV-infected patients.

HCV infection induced increase of MPV score but HIV infection resulted in its decrease. Thus, these two opposite trends may counteract reciprocally during HIV/HCV coinfection and lead to no significant change of MPV. This data indicated that, it is not appropriate to conclude that coinfection with HCV would further aggravate the decreased MPV induced by HIV infection, or that coinfection with HIV would improve the increased MPV caused by HCV infection. Although MPV could be a potential indicator of platelet activation and function, and even of hepatic fibrosis^22,33^, evidences are lack on its meaning in the condition of HIV/HCV coinfection. Higher risk of CVD events in HIV infection may have little association with greater platelet reactivity as identified by MPV. On the other hand, both HIV and HCV viruses were associated with reduction of platelet counts, indicating that coinfection with HCV would aggravate symptom of thrombocytopenia induced by chronic HIV infection, and link to an increased risk of CVD in HIV-infected individuals. Though MPV scores were unchanged in coinfected patients, it is possible that the activity and function of platelets were impaired, which need to be further studied in the future.

In all, HCV coinfection may aggravate the decrease of platelet counts, but not MPV score in chronic HIV infection. MPV showed poor applicability in reflecting the status of platelet in HIV/HCV-coinfected patients.

## Methods

### Study design and participants

A longitudinal survey of 318 individuals from a village of Anhui province was initiated in 2006. All recruited individuals were negative for hepatitis B surface antigen (HBsAg) and had never received any forms of HCV-specific antiviral therapy. Patients were also excluded if they had nonalcoholic and alcoholic steatohepatitis, autoimmune liver diseases, or hereditary and metabolic liver diseases.

**Figure 5:**
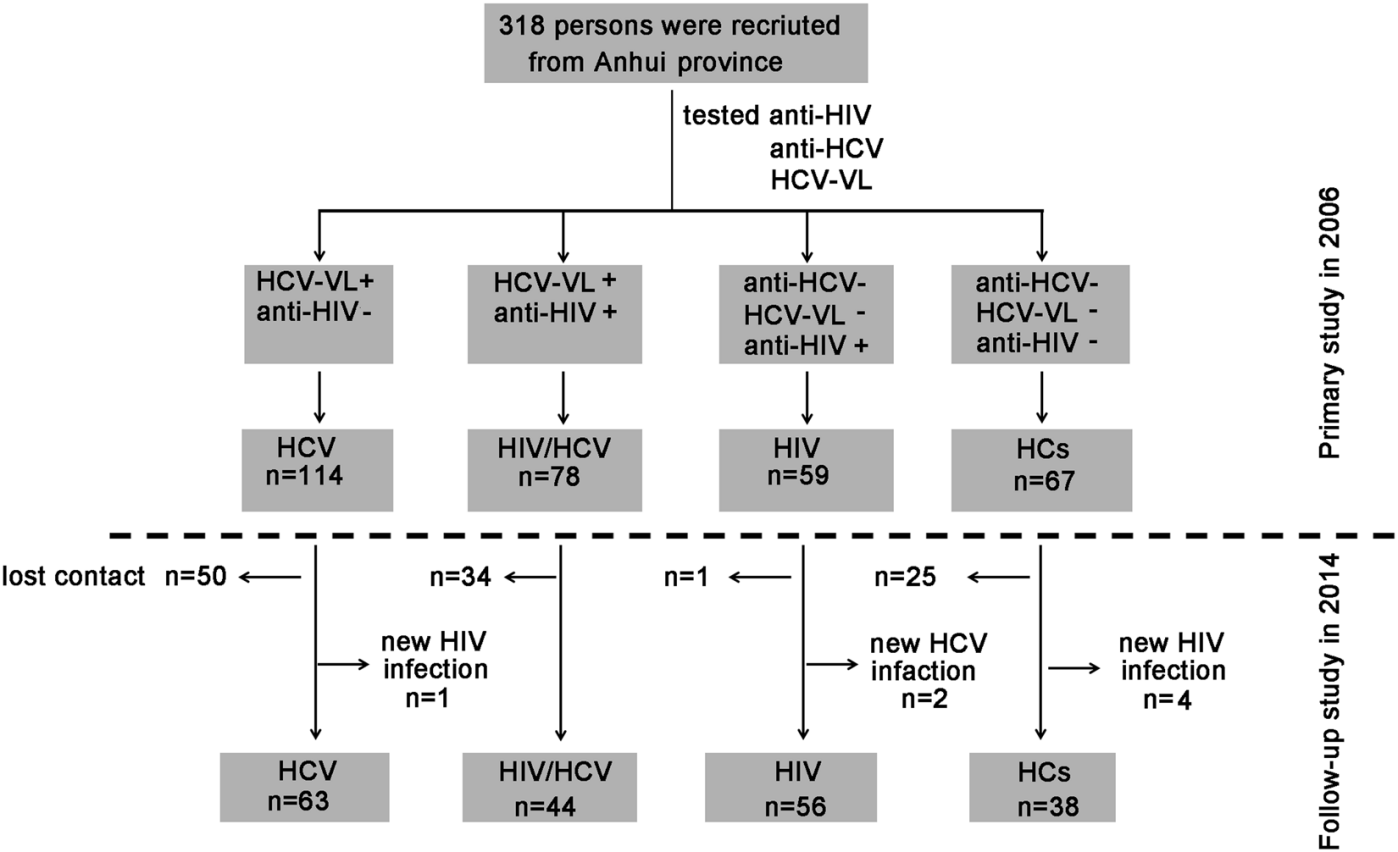
A flow diagram for construction of recruited individuals from primary visit in 2006 and secondary visit in 2014 in a village of Anhui province.

According to the performance of plasma anti-HIV, anti-HCV and HCV viral laod (HCV-VL) quantitation, four groups were included: HCV group (HCV-VL^+^ & anti-HIV^−^, n = 114), HIV/HCV group (HCV-VL^+^ & anti-HIV^+^, n = 78), HIV group (anti-HCV^−^ & HCV-VL^−^ & anti-HIV^+^, n = 59) and healthy controls (HCs) group (anti-HCV^−^ & HCV-VL^−^ & anti-HIV^−^, n = 67). Of the 318 recurited subjects, 201 (HCV group, n = 63; HIV/HCV group, n = 44; HIV group, n = 56; HCs group, n = 38) were successfully followed up in 2014 and retested for HCV-VL and anti-HIV. Individuals with new infection of HCV or HIV were excluded, and 117 subjects lost follow-up due to death or loss of contact. The clinical backgrounds of the recruited individuals in 2006 were shown in Table S1. Also, we compared the basic characteristics between individuals who were successfully followed in 2014 and who were lost contact in 2014, and found no significant difference (Table S2). A flow diagram for recruited subjects was illustrated in Fig. 5.

Most of these HCV-infected and HIV-infected patients had unsanitary commercial blood collection practices until the end of the 1990’s. All HIV-infected patients had previously received regular or intermittent HIV-specific antiretroviral therapy (ART) consisting of two nucleoside reverse transcriptase inhibitors (NRTIs): azidothymidine (AZT) plus didanosine (ddI); or stavudine (d4T) plus lamivudine (3TC), and one non-nucleoside reverse transcriptase inhibitor (NNRTI): nevirapine (NVP). The treatment was provided through support from the China CARES (Community AIDS Resource and Education Services) program. All participants were interviewed by trained and qualified staff using a standardized questionnaire, including detailed general information, blood donation history, and usage of antiviral or antiretroviral drugs.

A fasted venous blood sample was collected from each participant between 7~8 am after an overnight fast of 8~12 h. All complete blood count (CBC) analyses were performed in the clinical laboratory of the local CDC. CBC analysis was performed using a Gen-S automated analyzer (Beckman Coulter, High Wycombe, UK) within 2 h after sample collection. Serum liver function indexes were measured by traditional clinical standardized methods on UnicerDxc 800 Synchron Clinical System (Beckman Coulter, Fullerton, CA, USA). Plasma HCV-RNA measurement was performed using the Abbott Real-Time HCV Amplification Kit (Abbott Molecular Inc. Des Plaines, IL, USA) according to the manufacturer’s instructions. Plasma HCV antibody was detected using the Abbott Architect anti-HCV assay (Abbott GmbH & Co KG, Wiesbaden, Germany). Additionally, all reagents used for CD4^+^/CD8^+^ T-cell counts were purchased from BD Biosciences (BD Biosciences, San Jose, CA) and CD4^+^/CD8^+^ T-cell counts were measured within 12 hours using a FACS Calibur (BD Biosciences, San Jose, CA).

### Statistical analysis

Descriptive statistics were shown as mean and standard deviation, as appropriate. Spearman’s rank-correlation, Wilcoxon matched-pairs and Mann-Whitney U-tests were performed using GraphPad Prism 5.0 software (GraphPad Software Inc., San Diego, CA) when necessary. In some circumstances, receiver operating characteristic (ROC) and the logistic regression analysis was also performed by SPSS (Version 21.0, SPSS, Inc., Chicago, IL). All p-values were two-tailed, and were considered significant when lower than 0.05.

### Ethical approvals

All subjects provided written informed consent to participate in the study. The study and all methods were approved by the institutional review authorities of Peking University Health Science Center (Approval ID: PKUPHLL20090011). All methods were performed in accordance with the relevant guidelines and regulations.

## Electronic supplementary material

Supplementary data

## Data Availability

All data generated or analyzed during this study are included in this manuscript.
