# Supplementary material for: HCV coinfection aggravated the decrease of platelet counts, but not mean platelet volume in chronic HIV-infected patients
Source: Sci Rep. 2018 Nov 30;8:17497. doi: 10.1038/s41598-018-35705-9 (PMC6269489; doi:10.1038/s41598-018-35705-9)
Supplement: Supplementary file 1 — Supplementary data [file 41598_2018_35705_MOESM1_ESM.docx]

**Supplementary information**

**Manuscript title:**

HCV coinfection aggravated the decrease of platelet counts, but not mean platelet volume in chronic HIV-infected patients

**Authors:**

Linting Lv^1,#^, Yuantao Li^1,#^, Xueying Fan^1^, Zhe Xie^1^, Hua Liang^2,*^, Tao Shen^1,*^

^1^Department of Microbiology and Center of Infectious Disease, School of Basic Medical Sciences, Peking University Health Science Center, Beijing 100191, China.

^2^State Key laboratory of Infectious Disease Prevention and Control (SKLID), National Center for AIDS/STD Control and Prevention, China CDC, Collaborative Innovation Center for Diagnosis and Treatment of Infectious Diseases, Beijing 102206, China.

^#^These authors contributed equally to the manuscript.

*Corresponding authors:

Tao Shen, MD & PhD

Department of Microbiology and Center of Infectious Diseases, Peking University Health Science Center, 38 Xueyuan Road, Haidian District, Beijing, 100191, China

Tel: 86-10-82805070; Fax: 86-10-82805136

Email: taoshen@hsc.pku.edu.cn

Hua Liang, MD & PhD

State Key laboratory of Infectious Disease Prevention and Control (SKLID), National Center for AIDS/STD Control and Prevention, China CDC, Collaborative Innovation Center for Diagnosis and Treatment of Infectious Diseases, Beijing 102206, China.

Email: lianghua@chinaaids.cn

Table S1. The clinical characteristics of individuals enrolled in 2006 in this study.

| Characteristics | HIV/HCV | HCV | HIV | HCs |
| --- | --- | --- | --- | --- |
| Case (N) | 78 | 114 | 59 | 67 |
| Gender (M/F) | 35/43 | 52/62 | 25/34 | 16/51 |
| Age (y)* | 44.21(9.82) | 49.91(12.80) | 43.41(10.24) | 46.19(13.00) |
| BMI* | 22.02 (1.31) | 23.14 (1.54) | 23.31 (1.28) | 23.09 (1.40) |
| HBsAg | - | - | - | - |
| Anti-HIV | + | - | + | - |
| HCV-VL (log^10^IU/ml)* | 6.01(0.9) | 6.39(0.79) | - | - |
| Anti-HCV (S/CO)* | 13.34(2.80) | 14.46(1.89) | - | - |
| CD4+T-cell(cells/μl)* | 432.57(210.58) | 851.39(327.85) | 467.26(282.66) | 914.97(354.67) |
| CD8+T-cell(cells/μl)* | 1045.65(628.22) | 666.14(348.27) | 981.78(360.33) | 717.42(361.05) |
| HCVgenotype,n (%) |  |  |  |  |
| 1b | 64 | 67 | N.A. | N.A. |
| 2a | 36 | 33 | N.A. | N.A. |
| Others | 0 | 0 | N.A. | N.A. |
| Biochemistry analysis |  |  |  |  |
| ALT(IU/L)* | 49.14 (48.12) | 47.48(34.61) | 30.30(23.43) | 20.22(13.64) |
| AST(IU/L)* | 52.15 (43.78) | 42.81(21.39) | 36.25(22.49) | 24.78(10.27) |
| Total protein (g/l) | 79.14(7.00) | 76.33(8.21) | 77.46(6.23) | 75.44(5.89) |
| Albumin (g/l) | 44.21(8.85) | 44.36(5.85) | 44.66(6.80) | 42.41(5.65) |
| TBil (μmol/l), | 13.88(7.75) | 13.76(3.51) | 13.61(3.90) | 13.44(3.38) |
| DBil (μmol/l) | 4.28(2.55) | 4.56(2.53) | 4.45(1.64) | 4.59(2.30) |
| Blood rountine |  |  |  |  |
| RBC(×10^12^/L)* | 4.14 (1.42) | 6.11(0.33) | 3.99(1.21) | 6.16(0.41) |
| WBC(×10^9^/L)* | 5.92(2.42) | 13.15(2.47) | 4.39(2.69) | 3.65(0.07) |
| GRA%* | 60(19) | 59(19) | 62(10) | 42(2) |
| LYM%* | 38(10) | 39(10) | 38(10) | 58(2) |

*, mean+standard deviation; HBsAg, HBV surface antigen; HIV, human immunodeficiency virus; HCV, hepatitis C virus; VL, viral load; ALT, alanine aminotransferase; AST, aspartate aminotransferase; TBil, total bilirubin; DBil, direct bilirubin; GRA, granulocyte; LYN, lymphocyte; BMI, body mass index, calculated as the weight in kilograms divided by the square of height in meters; N.A., not available.

Table S2. Comparison of basic characteristics between individuals who were followed in 2014 and individuals who were lost contact in 2014.

| Characteristics in 2006 | Followed in 2014  (n=201) | Lost contact in 2014  (n=117) | P value |
| --- | --- | --- | --- |
| Female (n, %) | 132, 65.67 | 72, 61.54 | 0.4693 |
| Age (year)* | 47.17±0.67 | 46.97±1.198 | 0.8762 |
| PLT (×10^9^/L) * | 209.7±6.80 | 230.3±13.39 | 0.1411 |
| MPV(fl) * | 10.97±0.21 | 11.09±0.10 | 0.6498 |
| CD4+T-cell(cells/μl)* | 711.80±26.42 | 645.60±29.27 | 0.0986 |

*, mean±standard deviation; PLT, platelet; MPV, mean platelet volume. The distribution of gender between two groups was compared by the chi-square test and the other characteristics were compared by unpaired t test.

Table S3. Univariate logistic regressions analyses of variables associated with different prognosis of anti-HIV therapy in 2014.

| Variables in 2006 | Odds ratio | 95% confidence interval | P value |
| --- | --- | --- | --- |
| PLT (×10^9^/L)* | 1.035 | (0.330, 3.244) | 0.953 |
| MPV(fl)* | 1.669 | (0.483, 5.773) | 0.418 |
| Age (year) | 2.864 | (0.641, 12.798) | 0.168 |
| Gender | 0.975 | (0.297, 3.201) | 0.966 |
| HCV infection | 0.360 | (0.093, 1.390) | 0.138 |

*Receiver operating characteristics (ROC) analysis was performed to determine the cutoff points for PLT and MPV to predict the status of CD4-T cell count in HIV-monoinfected patients. All variables were applied to discretized or digitized prior to logistic regression analysis: “1” = “PLT > 185.5” and “0” = “ PLT≤185.5”; “1” = “MPV > 10.45” and “0” = “MPV ≤10.45”; “1”=“ > 50 year old” and “0” = “≤ 50 year old”; “1” = “male” and “0” = “female” for gender; “1” = “HIV/HCV coinfection” and “0” = “HIV monoinfection”; “1” = “CD4+ T-cell ≥500/µl” in 2014 and “0” = “CD4+ T-cell < 500/µl” in 2014.

Figure S1

**
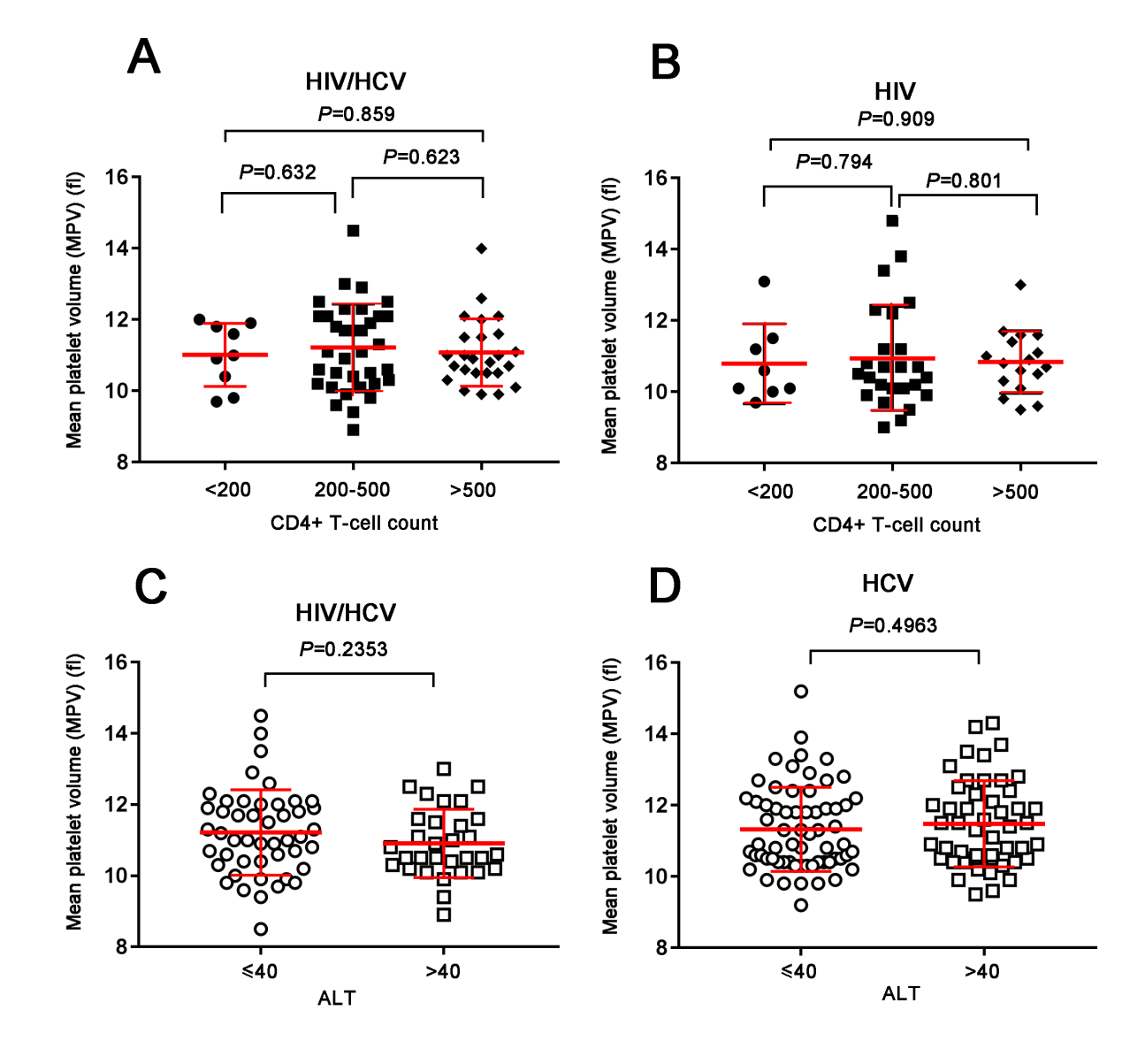
**

**Figure S1. Comparison of MPV scores among subgroups of patients infected with HCV and HIV separately or jointly.** HIV/HCV (**A**) and HIV (**B**) groups were divided into three subgroups according to CD4+ T-cell count (<200/μl, 200-500/μl and >500/μl). Similarly, HIV/HCV (**C**) and HCV (**D**) groups were divided into two subgroups according to serum ALT level (≤40 IU/L and> 40 IU/L). Bars indicated mean ± standard deviation for each subgroup. *P*-values were calculated by Mann-Whitney *U* test.
